# Evolution post-opératoire des séquelles de tuberculose pulmonaire chez les séropositifs VIH

**DOI:** 10.11604/pamj.2014.17.20.3424

**Published:** 2014-01-17

**Authors:** Ayegnon Kouakou Grégoire, Kendja Hypolite Flavien, Ouédé Raphaël, Blaise Démine, Ménéas Gueu Christophe, Ano Kounangui Marie, Yangni-Angaté Koffi Hervé, Tanauh Yves

**Affiliations:** 1 Service des Maladies Cardio-Vasculaires et Thoraciques du CHU de Bouaké; 2 Service de Chirurgie Thoracique de l'ICA

**Keywords:** VIH/SIDA, séquelles, tuberculose pulmonaire, chirurgie, evolution, HIV/AIDS, sequelae, pulmonary tuberculosis, surgery, evolution

## Abstract

Cette étude rapporte les aspects cliniques et évolutifs des séquelles pulmonaires tuberculeuses (SPT) opérées chez les séropositifs (VIH+). Il s'agit d'une étude prospective transversale réalisée entre Novembre 2005 et Octobre 2012. Elle a porté sur 20 patients VIH+, ayant dans leurs antécédents, une tuberculose pulmonaire (TP) traitée et déclarée guérie, et admise dans ladite période pour une chirurgie de la SPT secondaire. Une enquête sérologique VIH a été réalisée systématiquement au cours du bilan pré-opératoire. Le diagnostic pré-opératoire de la SPT, la mortalité, les complications post- opératoires (CPOP), le séjour hospitalier, le suivi à moyen terme des STP opérées ont été évalués. Les séropositifs étaient VIH1+ (n = 12; 60%), VIH1&2+ (n = 4; 20%) et VIH2+ (n = 4; 20%). La durée moyenne d’évolution des STP était de 26,22 ± 21,3 mois. Les STP étaient les pyothorax ou pleurésies enkystées (n = 16; 80%), le poumon détruit (n = 2;10%) et les dilatations de bronches (n = 2;10%). Les VIH^+^ ne présentaient pas d'aspergillome pulmonaire. Le séjour hospitalier moyen était 13,1 ± 10,2 jours. Le suivi total était de 82 patients-année avec une moyenne de suivi de 4,2 ± 2,3 ans (extrêmes: 1 et 7 ans). Le taux de mortalité à court et moyen terme était nul. Aucun décès post-opératoire immédiat n'a été noté. Les CPOP immédiates étaient les bullages prolongés chez 75% des immunodéprimés. Les CPOP tardives (n = 3) étaient un syndrome restrictif pulmonaire, un pyothorax persistant et une pachypleurite résiduelle restrictive. A court terme, le taux de guérison radiologique était de 80% (n = 16).

## Introduction

Avec l′épidémie du Virus de l′Immunodéficience Humaine (VIH), faisant de la tuberculose pulmonaire la première maladie opportuniste du SIDA depuis 1993 [1, 2]; l′évolution des séquelles de la tuberculose pulmonaire (STP) opérées connait un regain d′intérêt. Quant aux résultats chirurgicaux de ces STP des séropositifs VIH (VIH^+^), des interrogations demeurent toujours. Ainsi avons nous entamé cette étude dans le but de bien appréhender les différentes formes cliniques des STP chez les VIH^+^ et de connaitre leur évolution post-opératoire.

## Méthodes

Entre Novembre 2005 et Octobre 2012, nous avons réalisé au service de Chirurgie thoracique de l'Institut de Cardiologie d'Abidjan, une étude prospective transversale portant sur 20 patients séropositifs VIH ayant bénéficié d'un geste chirurgical pour une STP symptomatique. Tous les patients avaient dans leurs antécédents, une TP traitée et déclarée guérie, et admises dans ladite période pour une chirurgie de la STP secondaire. Une enquête sérologique VIH a été réalisée systématiquement au cours du bilan pré-opératoire. Aucune enquête environnementale complémentaire n'a été réalisée. La radiographie pulmonaire et le scanner thoracique ont été contributifs au diagnostic dans tous les cas. Le diagnostic pré-opératoire retenu de la STP, la mortalité, les complications post-opératoires, le séjour hospitalier, le suivi à moyen terme des STP opérées ont été évalués. Les variables continues ont été exprimées en moyenne ± Erreur standards. Les probabilités ont été exprimées en pourcentages ± l'intervalle de confiance à 95%. La survie et les complications liées à la chirurgie ont été décrites par la méthode de Kaplan-Meier. Le seuil de significativité a été fixé à p < 0,05.

## Résultats

**Figure 1:**
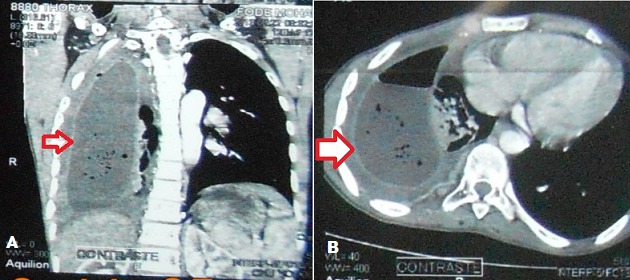
A)Pyothorax chez 1 séropositif VIH+; B) Pyothorax chez 1 séropositif VIH+

**Figure 2:**
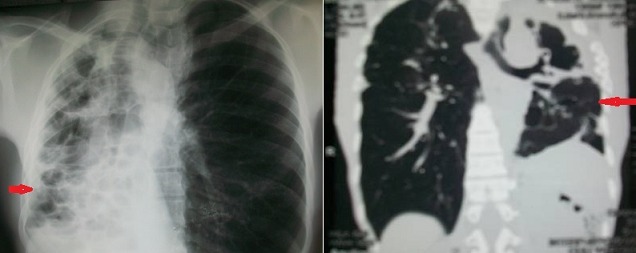
A) Poumon détruit chez des séropositifs VIH+;B) Poumon détruit chez des séropositifs VIH+

**Figure 3:**
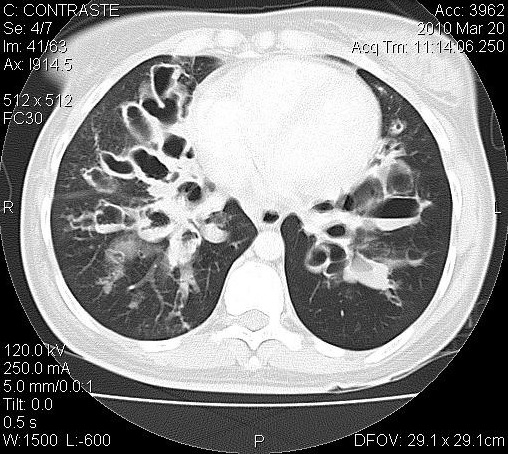
Dilatation de bronches de localisation bilatérale chez 1 VIH+

**Figure 4:**
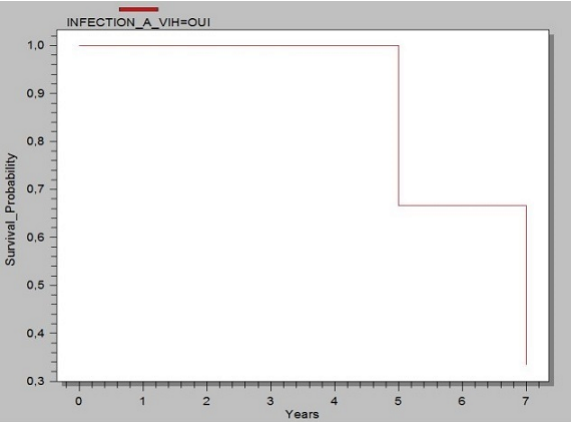
Courbe de survie globale de Kaplan –Meier en l'absence de complications post –opératoires de STP (excluant les bullages prolongés)

Il s'agissait de 20 patients séropositifs VIH qui ont été consulté pour une STP. L’âge moyen était de 39,4 ± 6,91. On notait 10 Hommes (H) (50%) et 10 Femmes (F) (50%), soit un sex-ratio (H/F) de 1. La séropositivité était de type VIH1^+^ (n = 12; 60%), VIH 1&2^+^ (n = 4; 20%) et VIH 2^+^ (n = 4; 20%). 10 patients VIH 1^+^(p = 0,625; IC95 = 0,245- 0,915; p = S), 4 patients VIH 2^+^ et 2 patients VIH1&2^+^ souffraient d'un pyothorax. 2 patients VIH1^+^ avaient un lobe ou un poumon détruit et 2 patients VIH1&2^+^ , une dilatation de bronche. Les caractéristiques diagnostiques et les gestes chirurgicaux réalisés ont été récapitulés dans le Tableau 1. Tous les patients présentaient une séquelle radiologique de la TP. Le délai médian de consultation en chirurgie thoracique après un traitement antituberculeux était de 2,5 ans (n = 17 patients). Au plan diagnostique, les principales STP étaient le pyothorax ou pleurésie enkystée (n = 12; 60%) Figure 1, le poumon détruit (n = 2; 10%) Figure 2 et la dilatation de bronches dont une localisation bilatérale (n = 2; 10%) (Figure 3). Au moment de la chirurgie, 90% des patients VIH^+^ (n = 18) étaient sous traitement anti-retroviral (ARV). Chez ces patients sous le traitement ARV, le taux médian d'anticorps CD4 était de 182,5 UI (n = 16). Ce taux d'anticorps CD4 était inférieur à 150 UI (n = 8), compris entre 150 UI et 200 UI (n = 4) et entre 250 UI et 300 UI (n = 4). Chez les 2 autres patients, le traitement ARV était institué précocement au début de l'infection VIH/SIDA. 10% des patients n’étaient pas sous traitement ARV. Le taux médian d'anticorps CD8 était de 1802 UI (n = 6). Par thoracotomie postéro-latérale avec ou sans épargne musculaire, nous avons réalisé: 2 pneumonectomies, une bilobectomie associée à une thoracoplastie; 3 lobectomies; 10 décortications pleurales qui se situaient toutes à droite et 4 drainages pleuraux. La distribution des gestes chirurgicaux a été illustrée au Tableau 2. On a réalisé 6 résections pulmonaires (30%). La mortalité immédiate était nulle. Le séjour moyen hospitalier était 13,1 ± 10,2 jours. Le suivi total était de 82 patients-année avec une moyenne de suivi de 4,2 ± 2, 3 ans (extrêmes: 1 et 7 ans). Le taux de complications post-opératoires immédiates était de 75% (n = 15) dues aux bullages prolongés. Le taux de complications tardives 15% (n = 3) due à un syndrome restrictif pulmonaire (n =1), une pachypleurite restrictive (n = 1) et un pyothorax droit persistant (n = 1), reconstitué totalement à 2 ans post-opératoires. La survie globale sans complications post- opératoires des STP des immunodéprimés est illustrée par la courbe de Kaplan-Meier Figure 4. A court terme, le taux de guérison radiologique était de 80% (n = 16).

**Tableau 1:** Caractéristiques démographiques et cliniques des séropositifs opérés

Caractéristiques démographiques et cliniques	Tranche d’âge (ans)	VIH ^+^	
		Effectifs (n)	Pourcentage (%)
AGE (ans)	< 20	0	0
	20 - 30	2	10
	30 - 40	6	30
	40 – 50	12	60
	50 – 60	0	0
	60 – 70	0	0
	**TOTAL**	**20**	**100**
Sexe	Masculin (M)	10	50
	Féminin (F)	10	50
Antécédents	1 épisode de TP	14	70
	1er récidive	2	10
	2em récidive	4	20
Durée moyenne de la maladie (STP)	26,22 ± 21, 3 mois (n = 18)		
Diagnostic des séquelles de tuberculose pulmonaire (STP)	Poumon détruit (D/G)	2	10
	Dilatation de Bronches	2	10
	Aspergillome pulmonaire	0	0
	Pyothorax ou Pleurésie enkystée	16	80

**Tableau 2:** Les actes chirurgicaux chez les séropositifs VIH

Actes chirurgicaux		Effectifs (n)	Pourcentage (%)
**Type de Chirurgie**	Chirurgie sans résection pulmonaire	14	70
	Résection lobaire (lobectomie/bilobectomie)	4	20
	Résection pulmonaire complète (pneumonectomie)	2	10
**Gestes chirurgicaux précis**	Bilobectomie	1 (1D)	5
	Décortication pleurale	10 (10D)	50
	Drainages pleuraux	4 (4D)	20
	Lobectomie supérieure	2 (2D)	10
	Lobectomie moyenne	0 (-)	0
	Lobectomie inférieure	1 (1D)	5
	Pneumonectomie	2 (2G)	10

## Discussion

Au-delà du profil épidémiologique déjà connu du couple VIH/TP, l’évolution de la STP chez le séropositif VIH, constitue toujours un paradigme tant pour les médecins que pour les malades [2–4]. C'est pourquoi nous nous intéressons dans ce travail aux aspects cliniques et l’évolution post opératoire des STP. Dans notre travail, la tranche d’âge de prédilection des STP opérées était de 50 à 60 ans chez les séropositifs au VIH^+^ . Ce qui concorde avec les données de la littérature sur le VIH [3, 5, 6]. A postéori, l'on s'attendait à une prédominance de STP chez les femmes VIH+, Mais la prédominance féminine des séropositifs VIH en Afrique n'est pas retrouvée en cas de STP. Dans notre étude, les patients VIH^+^ ne présentaient pas de STP à type d'aspergillome pulmonaire alors que 30% de nos patients (n = 6) consultèrent pour une séquelle fonctionnelle de TP à type d'hémoptysie. Ce constat concorde avec la rareté des lésions cavitaires et une localisation apicale moins fréquente de la TP chez les personnes vivant avec le VIH (PVVIH) [7, 8]. L'absence d'aspergillome pulmonaire dans notre série est contraire aux résultats de JL. Rokotoson et al. [9] qui ont révélé 1 cas d'aspergillome pulmonaire chez un VIH^+^ dans une série de 37 STP. Dans notre travail, la plupart des patients VIH^+^ avaient des pyothorax ou pleurésies enkystées. Cela était probablement en rapport avec leur grande susceptibilité à l'infection. La mortalité post-opératoire immédiate chez les immunodéprimés était nulle comparable à celle d'une chirurgie thoracique simple [10]. Dans une étude similaire, JL. Rokotoson et al. [9] montraient que la co-infection VIH/STP représentait 2,7% et que la chirurgie de résection pulmonaire entraînait 4% de décès. En effet, l'absence de décès immédiat pourrait s'expliquer par le fait que 90% des patients VIH^+^ étaient sous ARV avec une profondeur modérée de leur immunodépression. Mais il faudrait craindre, selon K. Cohen [11], la co-toxicité du traitement anti-rétroviral et anti-tuberculeux compromettrait les résultats chirurgicaux escomptés de par la néphro-toxicité et l'hépato-toxicité qu'occasionne cette association thérapeutique. Comme dans notre étude, A. Harries et al. [12] ont suggéré le traitement ARV en pré et post-opératoire des STP chez les immunodéprimés.

Tout comme les immunocompétents, les séropositifs au VIH ayant une séquelle fonctionnelle de la TP à type d'une hémoptysie en pré-opératoire ou d'un bullage prolongé post-opératoire, sont à risque de complications post-opératoires à moyen terme [7–13]. Dans notre série 3 séropositifs sur 4 avaient un bullage prolongé alors que 1 sur 5 malades a bénéficié d'une résection partielle ou totale du poumon. La forte prédisposition aux bullages prolongés post-opératoires n'est donc pas l'apanage de la résection pulmonaire chez le séropositif VIH opéré d'une STP. La décortication pleurale des VIH^+^ augmenterait dans l'immédiat la fréquence des bullages prolongés post opératoires des STP. A court et moyen terme, le taux de CPOP des STP chez l'immunodéprimé est superposable à celui des immunocompétents [4, 10, 13].

## Conclusion

Le statut VIH^+^ ne constitue pas un frein à l'indication chirurgicale des STP. Sous une trithérapie rétrovirale précoce; l’évolution post-opératoire immédiate de ces STP chez le séropositif VIH est marquée par des bullages prolongés. A court terme la guérison radiologique est satisfaisante. A court et moyen terme, la morbidité des STP opérées chez les immunodéprimés est sans particularité.
